# Mortality in a cohort of remote-living Aboriginal Australians and associated factors

**DOI:** 10.1371/journal.pone.0195030

**Published:** 2018-04-05

**Authors:** Zoë Hyde, Kate Smith, Leon Flicker, David Atkinson, Osvaldo P. Almeida, Nicola T. Lautenschlager, Anna Dwyer, Dina LoGiudice

**Affiliations:** 1 Western Australian Centre for Health and Ageing, Centre for Medical Research, University of Western Australia, Perth, Australia; 2 School of Medicine and Pharmacology, University of Western Australia, Perth, Australia; 3 Rural Clinical School of WA, University of Western Australia, Perth, Australia; 4 Kimberley Aboriginal Medical Services, Broome, Australia; 5 School of Psychiatry and Clinical Neurosciences, University of Western Australia, Perth, Australia; 6 Academic Unit for Psychiatry of Old Age, Department of Psychiatry, University of Melbourne, Melbourne, Australia; 7 NorthWestern Mental Health, Melbourne Health, Melbourne, Australia; 8 Nulungu Research Institute, University of Notre Dame, Broome, Australia; 9 Aged Care, Melbourne Health and University of Melbourne, Melbourne, Australia; Australian National University, AUSTRALIA

## Abstract

**Objectives:**

We aimed to describe mortality in a cohort of remote-living Aboriginal Australians using electronic record linkage.

**Methods:**

Between 2004 and 2006, 363 Aboriginal people living in remote Western Australia (WA) completed a questionnaire assessing medical history and behavioural risk factors. We obtained mortality records for the cohort from the WA Data Linkage System and compared them to data for the general population. We used Cox proportional hazards regression to identify predictors of mortality over a 9-year follow-up period.

**Results:**

The leading causes of mortality were diabetes, renal failure, and ischaemic heart disease. Diabetes and renal failure accounted for 28% of all deaths. This differed from both the Australian population as a whole, and the general Indigenous Australian population. The presence of chronic disease did not predict mortality, nor did behaviours such as smoking. Only age, male sex, poor mobility, and cognitive impairment were risk factors.

**Conclusions:**

To reduce premature mortality, public health practitioners should prioritise the prevention and treatment of diabetes and renal disease in Aboriginal people in remote WA. This will require a sustained and holistic approach.

## Introduction

Despite living in one of the world’s wealthiest countries, Indigenous Australians have a life expectancy lower than that of indigenous peoples living elsewhere [1] and about 10 years lower than non-Indigenous Australians [2]. In contrast to what takes place in many developing nations, this decrease in life expectation is attributable to high rates of premature death rather than infant mortality [2–4]. In response to these observations, the Australian Government set six key targets for Indigenous health and well-being, including closing the mortality gap within a generation [5]. The causes of the life expectancy gap are most likely multifactorial, and seem to include factors such as obesity, physical inactivity, smoking, alcohol abuse and poor nutrition [6, 7]. These risk factors are associated with socioeconomic disadvantage and psychosocial stressors, including intergenerational trauma, lower educational attainment, higher unemployment, lower income, overcrowded living conditions, poor community infrastructure, and barriers that limit access to health services [3, 7, 8].

Aboriginal and Torres Strait Islander Australians comprise approximately 3% of Australia’s population, most of whom (79%) live in urban or regional Australia. Only one-fifth live in remote or very remote areas [2]. However, those living in remote Australia account for 40% of the life expectancy gap [9]. We conducted this study to explore how health outcomes might differ in remote areas, with a view to better inform public health efforts to reduce premature mortality. In this cohort study, we aimed to: (i) describe the leading causes of mortality in a remote-living Aboriginal population; (ii) determine predictors of all-cause mortality; and, (iii) identify causes of death in excess of what would be expected for the general population, and describe potentially preventable causes of mortality.

## Methods

### Study population

Between 2004 and 2006, we recruited 363 Aboriginal Australians living in the remote Kimberley region of Western Australia (response fraction: 94.3%). Participants were aged 45 years or older at study entry, and were recruited from the communities of Ardyaloon, Junjuwa, Looma, Mowanjum, Warmun, and Wirrimanu, and from the town of Derby. To construct the sampling frame, we were helped by local Aboriginal health and community services to identify communities (and one town) which were representative of the Aboriginal peoples of the Kimberley region, based on the 5 major language families of this area. People who were resident in the identified communities for at least 6 months of the year were approached, together with a random sample of one-third of eligible people living in Derby. Full details of the study protocol have been published elsewhere [10]. The age distribution of the sample was similar to that of the Australian population as a whole, for those over 45 years [11].

### Study design

Research assistants administered culturally-appropriate questionnaires to participants and their family members and/or carers. The questionnaire assessed self- and family-reported medical history, use of tobacco and alcohol, cognition, mood, and activities of daily living. The questionnaire and related material is available from: http://www.wacha.org.au/

We used electronic record linkage to capture endpoints (ICD-10 coded cause of death). Data linkage was performed by the Data Linkage Branch (DLB) of the Western Australian Department of Health. The DLB manages Western Australia’s population datasets, which include all deaths registered in the state [12, 13]. Death records were available to the end of July 2013, but 20 deaths had not yet been ICD-coded by the Australian Bureau of Statistics (several months lag is usual in this regard). However, since the cause of death noted on the death certificate was available, these records were coded by ZH and DL. The DLB could not link one person, so analyses were restricted to the 362 people for whom data were available.

Community engagement and participation: Engagement and participation of the individual communities continued throughout the project in accordance with research guidelines [14]. Initial approvals were sought from individual community councils and their advice about ways of participation within the community were adopted. The survey was developed and validated in the local region using participatory methods [10]. Community members were employed as research assistants to help identify participants and to administer the survey with assistance of interpreters if required, overseen by AD (Aboriginal Project Officer) and KS. The methodology of participation was guided by local community members. Each local community member employed was trained in the use of the survey, and basic training on research methods and health standards were provided. Education and feedback meetings were undertaken in each community (including to Community Councils) at various stages, including at conclusion of the project to incorporate mortality data, and also to local service providers and stakeholders. Approvals were granted from the Western Australian Aboriginal Health Ethics Committee and the Kimberley Aboriginal Health Planning Forum, and both committees reviewed these findings prior to submission for publication. Following involvement with this study a number of local research assistants undertook further studies in health.

### Ethics

Approval to conduct this study was obtained from the communities involved; the Kimberley Aboriginal Medical Services Council; Kimberley Aged and Community Services; the Kimberley Aboriginal Health Planning Forum Research Subcommittee; the Human Research Ethics Committee of the University of Western Australia; the WA Aboriginal Health Ethics Committee; and the Department of Health WA Human Research Ethics Committee. All participants provided written informed consent.

### Statistical analysis

We analysed the data with the Stata statistical package, version 11.2 (StataCorp, College Station, Texas). Demographic and clinical data for participants are stratified by age group, and presented as the number and proportion of people who answered in the affirmative for a given variable. We used Pearson’s Chi square test and Fisher’s exact test (as appropriate), to investigate associations between age group and the variables of interest. Deaths were grouped into both broad (e.g., neoplasms) and specific (e.g., lung cancer) causes, by the ICD codes used by the Australian Bureau of Statistics [15, 16]. Reference data for the general population were also drawn from the same sources [15, 16], and tabulated against the data for the sample. Only the number and proportion of the sample dying from each cause is presented, along with the corresponding proportion for the general population. We did not perform statistical testing (e.g., binomial probability tests) or present confidence intervals owing to the small number of deaths in each category. To establish whether mortality was greater among participants than the general population, we performed indirect standardisation to obtain a standardised mortality ratio (SMR). We used the 2012 population as reference. Because we were comparing mortality over a 10-year period in the sample to a single year in the general population (we could not compare mortality for the same year owing to the modest size of the cohort and the small number of deaths that occurred in that year), we calculated a SMR for each year of follow-up, weighted the resulting SMRs by the number of person-years of follow-up time as a proportion of the total follow-up time, and then summed the weighted SMRs to obtain a final estimate. We assessed predictors of all-cause mortality with Cox proportional hazards models. All demographic and clinical data were initially entered into univariate models, after which we entered variables that were significant in univariate analyses into a multivariable model, and then removed non-significant covariates in a manual, backwards manner. There were 22 people who had missing data for at least one item in the final multivariable model, and these people were excluded from the analyses. The underlying timescale for the regression analyses was time until death (in years), or until the censor date of 31 July 2013 (the end of the period for which mortality data were available). Age was entered into the regression analyses as the age (in years) at the time of entry into the study. We assessed the Schoenfeld residuals to confirm the proportional hazards assumption. We considered *p*-values <0.05 statistically significant.

## Results

The demographic, lifestyle and clinical characteristics of the sample are shown in Table 1. The mean age of the cohort at baseline was 60.7±11.9 years (range 45–96 years), and the mean follow-up time was 6.8±2.2 years (range 0.1–9.0 years). A total of 100 participants (27.6%) died during follow-up. The average age at death for both sexes was 71.2±12.7 years (range 46–95 years), while it was 70.7±12.6 years for men, and 71.8±13.1 years for women.

**Table 1:** Demographic, lifestyle, and clinical characteristics of the cohort, measured at recruitment (2004–2006).

Characteristic	Age at baseline (years)				
	45–49 (n = 71) n (%)	50–59 (n = 123) n (%)	60–69 (n = 71) n (%)	70–79 (n = 67) n (%)	80+ (n = 30) n (%)
**Sex**					
Male	27 (38.0)	61 (49.6)	37 (52.1)	26 (38.8)	13 (43.3)
Female	44 (62.0)	62 (50.4)	34 (47.9)	41 (61.2)	17 (56.7)
Some formal schooling**	64 (90.1)	102 (82.9)	28 (39.4)	20 (29.6)	4 (13.3)
**Poor vision**^18^	48 (67.6)	69 (56.1)	40 (56.3)	35 (52.2)	16 (53.3)
**Poor hearing**^18^*	12 (16.9)	24 (19.5)	5 (7.0)	12 (17.9)	8 (26.7)
**Prior stroke**^19^*	5 (7.0)	11 (8.9)	4 (5.6)	14 (20.9)	1 (3.3)
**Diabetes**^19^	28 (39.4)	46 (37.4)	25 (35.2)	31 (46.3)	5 (16.7)
**Hypertension**^19^	24 (33.8)	44 (35.8)	32 (45.1)	25 (37.3)	8 (26.7)
**Heart problem**^19^*	12 (16.9)	26 (21.1)	5 (7.0)	12 (17.9)	3 (10.0)
**Kidney problem**^19^**	13 (18.3)	21 (17.1)	5 (7.0)	5 (7.5)	3 (10.0)
**Poor mobility**^19^**	21 (29.6)	38 (30.1)	29 (40.9)	36 (53.7)	15 (50.0)
**Recent fall**^20^*	13 (18.3)	17 (13.8)	15 (21.1)	21 (31.3)	5 (16.7)
**Head injury with loss of consciousness**^21^	38 (53.5)	58 (47.2)	29 (40.9)	34 (50.8)	13 (43.3)
**Cognitive impairment** (KICA-Cog ≤35)^1^**	7 (9.9)	15 (12.2)	28 (39.4)	31 (46.3)	25 (83.3)
**Drink alcohol**^35^**	43 (60.6)	56 (45.5)	24 (33.8)	10 (14.9)	0 (0.0)
**Smoke tobacco**^35^**	39 (54.9)	47 (38.2)	28 (39.4)	10 (14.9)	2 (6.7)
Chew tobacco**	21 (29.6)	30 (24.4)	32 (45.1)	38 (56.7)	19 (63.3)
Died**	11 (15.5)	19 (15.5)	25 (35.2)	27 (40.3)	18 (60.0)

Note: Percentages calculated without excluding missing data (i.e., denominator is entire sample, n = 362), and are shown for columns.

Numerals in superscript denote number of people with missing data for that variable.

KICA-Cog = Kimberley Indigenous Cognitive Assessment tool.

Statistical testing was performed with either Pearson’s Chi square test or Fisher’s exact test, as appropriate;

* denotes *p* <0.05.

** denotes *p* <0.01.

Statistically significant differences were observed with regard to the three main behavioural risk factors assessed: alcohol use, smoking, and chewing tobacco. Alcohol and tobacco use was more common in younger age groups, while chewing tobacco was more common among older people. Chronic disease, particularly diabetes, hypertension and renal disease, was common. Mobility problems were common in all age groups, rising from almost one-third in the youngest age group to one-half among the oldest. Cognitive impairment was present in 11% of people aged 45–59 years, approximately 40% in those aged 60–79 years, and was ubiquitous in participants aged ≥80 years. Table 1

### Broad causes of mortality

Table 2 shows the underlying causes of death for the Australian population as a whole, and for this cohort. Mortality from neoplasms was lower than expected, accounting for only one-fifth of deaths compared to nearly one-third in the general population. Mortality from all circulatory system diseases, together with diseases of the respiratory system were also slightly less frequent than that of the general population, although some error is possible here due to the modest sample size. However, compared with the general community, mortality due to endocrine or metabolic diseases (17.0% vs. 4.1%) and diseases of the genitourinary system (14.0% vs. 2.6%) were far in excess of what would be expected.

**Table 2:** ICD-10 coded broad underlying causes of death in the Australian population as a whole, and in this study.

Cause of death	General population %	Study population n (%)
Certain infectious and parasitic diseases (A00-B99)	1.6	2 (2.0)
Neoplasms (C00-D48)	29.6	20 (20.0)
Diseases of the blood and blood-forming organs and certain disorders involving the immune mechanism (D50-D89)	0.3	0 (0.0)
Endocrine, nutritional and metabolic diseases (E00-E90)	4.1	17 (17.0)
Mental and behavioural disorders (F00-F99)	5.5	4 (4.0)
Diseases of the nervous system (G00-G99)	4.7	4 (4.0)
Diseases of the eye and adnexa (H00-H59)	<0.1	0 (0.0)
Diseases of the ear and mastoid process (H60-H95)	<0.1	0 (0.0)
Diseases of the circulatory system (I00-I99)	29.9	26 (26.0)
Diseases of the respiratory system (J00-J99)	9.0	6 (6.0)
Diseases of the digestive system (K00-K93)	3.6	2 (2.0)
Diseases of the skin and subcutaneous tissue (L00-L99)	0.3	0 (0.0)
Diseases of the musculoskeletal system and connective tissue (M00-M99)	0.8	0 (0.0)
Diseases of the genitourinary system (N00-N99)	2.6	14 (14.0)
Pregnancy, childbirth and the puerperium (O00-O99)	<0.1	0 (0.0)
Certain conditions originating in the perinatal period (P00-P96)	0.4	0 (0.0)
Congenital malformations, deformations and chromosomal abnormalities (Q00-Q99)	0.4	0 (0.0)
Symptoms, signs and abnormal clinical and laboratory findings, not elsewhere classified (R00-R99)	1.0	0 (0.0)
External causes of morbidity and mortality (V01-Y98)	6.3	5 (5.0)

Note: Cause of death data for the Australian population shown for the year 2012 [15].

### Leading causes of mortality

The top 20 leading causes of death in the Australian population, together with the corresponding figures for the cohort, are shown in Table 3. Unlike the general population, participants were more likely to die from diabetes (16.0% vs. 2.9%) or renal failure (12.0% vs. 1.8%). Combined, these two diseases accounted for 28.0% of all deaths. Mortality attributable to other leading causes of mortality was similar to the population as a whole. However, in this cohort of remote Aboriginal Australians, the top three causes of death were diabetes, renal failure, and ischaemic heart disease (compared with ischaemic heart disease, cerebrovascular disease, and dementia in the general population).

**Table 3:** ICD-10 coded top 20 leading causes of death in the Australian population as a whole, and corresponding proportions for this study.

Specific cause of death	General population %	Study population n (%)
Coronary heart diseases (I20–I25)	13.6	14 (14.0)
Cerebrovascular diseases (I60–I69)	7.3	8 (8.0)
Dementia and Alzheimer’s disease (F00–F03, G30)	7.0	4 (4.0)
Lung cancer (C33, C34)	5.5	7 (7.0)
Chronic obstructive pulmonary disease (J40–J44)	4.0	1 (1.0)
Diabetes (E10–E14)	2.9	16 (16.0)
Colorectal cancer (C18–C21)	2.8	0 (0.0)
Cancer, unknown, ill-defined (C26, C39, C76–C80)	2.4	2 (2.0)
Heart failure and complications and ill-defined heart disease (I50–I51)	2.4	1 (1.0)
Prostate cancer (C61)	2.1	0 (0.0)
Breast cancer (C50)	1.9	2 (2.0)
Influenza and pneumonia (J09–J18)	1.8	5 (5.0)
Renal failure (N17–N19)	1.8	12 (12.0)
Suicide (X60–X84)	1.7	0 (0.0)
Pancreatic cancer (C25)	1.7	1 (1.0)
Accidental falls (W00–W19)	1.4	0 (0.0)
Hypertensive diseases (I10–I15)	1.3	1 (1.0)
Cardiac arrhythmias (I47–I49)	1.2	0 (0.0)
Other ill-defined causes (R00–R94, R96–R99, I46.9, I95.9, I99, J96.0, J96.9, P28.5)	1.2	0 (0.0)
Leukaemia (C91–C95)	1.1	1 (1.0)

Note: Cause of death data for the Australian population shown for the year 2012 [16].

The all-cause mortality rate for participants was more than double that of the general population (SMR = 2.6; 95% CI 2.1, 3.1), as was the rate for ischaemic heart disease (SMR = 2.8; 95% CI 1.6, 4.7). Sufficient reference population data was not supplied to compare rates for deaths attributable to diabetes and renal failure.

### Predictors of all-cause mortality for older Indigenous Australians

Survival analyses for the cohort are shown in Table 4. In univariate models, age, male sex, poor mobility, a recent fall, chewing tobacco, and cognitive impairment were significant predictors of all-cause mortality. The risk associated with cognitive impairment was especially marked (HR = 4.5; 95% CI 3.0, 6.6). Having received some formal education was protective.

**Table 4:** Cox proportional hazards models showing associations between clinical and demographic factors at baseline and all-cause mortality for the Aboriginal study population.

Variable	Univariate	Age-adjusted	Multivariable
	HR (95% CI)	HR (95% CI)	HR (95% CI)
**Age** (years)	1.05 (1.03, 1.07)	-	1.03 (1.01, 1.05)
**Male sex**	2.08 (1.39, 3.11)	2.38 (1.58, 3.56)	2.17 (1.39, 3.39)
**Some formal schooling**	0.47 (0.32, 0.70)	0.90 (0.56, 1.45)	
**Poor vision**	1.14 (0.74, 1.76)	1.28 (0.83, 1.99)	
**Poor hearing**	1.29 (0.77, 2.16)	1.12 (0.66, 1.89)	
**Prior stroke**	0.92 (0.44, 1.91)	0.80 (0.39, 1.66)	
**Diabetes**	1.19 (0.76, 1.85)	1.18 (0.76, 1.83)	
**Hypertension**	1.34 (0.86, 2.10)	1.24 (0.79, 1.95)	
**Heart problem**	0.93 (0.52, 1.65)	1.00 (0.57, 1.78)	
**Kidney problem**	1.11 (0.62, 2.01)	1.22 (0.68, 2.21)	
**Poor mobility**	2.29 (1.49, 3.50)	1.87 (1.21, 2.88)	2.11 (1.34, 3.30)
**Recent fall**	1.84 (1.15, 2.93)	1.64 (1.03, 2.62)	
**Head injury with loss of consciousness**	0.95 (0.62, 1.45)	0.91 (0.60, 1.39)	
**Drink alcohol**	1.03 (0.65, 1.63)	1.80 (1.07, 3.03)	
**Smoke tobacco**	1.09 (0.69, 1.71)	1.62 (0.99, 2.65)	
**Chew tobacco**	1.49 (1.01, 2.21)	1.04 (0.68, 1.57)	
**Cognitive impairment**	4.46 (2.99, 6.64)	3.21 (2.01, 5.10)	2.19 (1.31, 3.65)

Note: Cognition was assessed with the Kimberley Indigenous Cognitive Assessment tool (KICA-Cog). Scores ≤35 were considered indicative of cognitive impairment.

Other than chewing tobacco, the behavioural risk factors that we assessed—being a current smoker (HR = 1.1; 95% CI 0.7, 1.7), and current alcohol use (HR = 1.0; 95% CI 0.7, 1.6)—were not associated with all-cause mortality. Having ever been a smoker (HR = 1.1; 95% CI 0.7, 1.8) or a drinker (HR = 1.3; 95% CI 0.8, 2.3) were also not significantly associated with mortality hazard (data for past substance use not shown in Table 4). Similar to current chewing of tobacco, having ever chewed tobacco was a significant predictor of mortality (HR = 1.62; 95% CI 1.03, 2.55). Medical comorbidity, such as diabetes, hypertension, cardiac problems and renal disease, was not significantly associated with all-cause mortality.

After adjustment, the only factors that remained significantly associated with all-cause mortality were age (HR = 1.03; 95% CI 1.01, 1.05), male sex (HR = 2.2; 95% CI 1.4, 3.4), poor mobility (HR = 2.1; 95% CI 1.3, 3.3), and cognitive impairment (HR = 2.2; 95% CI 1.3, 3.7). Five-year mortality risk by age group was as follows: 45–49 years, 11.3%; 50–59 years, 8.9%; 60–69 years, 21.1%; 70–79 years, 26.9%; ≥80 years, 43.3%. Kaplan-Meier survival curves are provided as supplementary material (S1 Fig).

## Discussion

In this cohort study of remote-living Aboriginal people, we observed a disproportionate number of deaths due to diabetes and renal disease compared with the general population. All-cause mortality was more than double that of the general population, and was high in all age groups. Even in the youngest age group (45–49 years) 5-year mortality was 11%. However, very few of the sociodemographic and clinical factors we measured were associated with mortality.

The only potentially modifiable risk factors associated with mortality were poor mobility and cognitive impairment. Both these factors could be indicative of premature ageing in this population, and Indigenous health policy is premised upon this idea, noting that many chronic diseases emerge at a younger age in Indigenous Australians than their non-Indigenous counterparts [17]. However, exactly what constitutes ageing is debatable [18], and the presence of diseases normally observed in old age does not necessarily imply that the ageing process is accelerated. Cotter and colleagues argue against the accelerated ageing thesis, based on cross-sectional analyses of population datasets and health survey data [19]. Rather than a uniform onset of geriatric syndromes, they found that only some age-associated conditions have an earlier onset in Indigenous Australians [19]. Alternatively, the association we observed between poor mobility and mortality could reflect a greater prevalence of frailty in this population [20]. Although frailty becomes more prevalent with age, it is not synonymous with ageing [21]. Frailty is a state of instability resulting from accumulated insults to multiple organ systems, ultimately leading to a failure of homeostasis and an increased risk of death and/or disability following a stressor event [22]. Physical and psychosocial stressors are common throughout the life course for many Aboriginal peoples [2, 3, 7]. This could suggest that unmeasured social determinants of health confer greater risk for mortality than behavioural factors, such as smoking and chewing tobacco.

Nonetheless, behavioural risk factors and medical comorbidity, including diabetes, renal disease, and hypertension, are almost certainly linked to premature mortality, being known risk factors for the leading causes of death observed in this cohort. The lack of statistical significance for these risk factors in our analysis may be attributable to under-reporting of health behaviours and/or recall bias, frequent under-diagnosis of chronic disease in remote Indigenous communities [23, 24], the relatively modest sample size and /or possible misclassification of deaths [25]. In addition, ubiquitous prevalence rates of diabetes, renal disease, and hypertension may minimise associations, and the resulting complications may lead to reported death outcomes [26]. However, our findings are similar to those of Burke and colleagues, who also conducted a longitudinal, data linkage study of Aboriginal people living in the Kimberley [27]. In their study, comprising 514 men and women aged 15–88 years, alcohol use (being an ex-drinker, or a current heavy drinker) was the only risk factor for all-cause mortality, while physical activity was protective. With regard to alcohol use in our study, the larger hazard ratio for having ever been a drinker, combined with the relatively wide confidence intervals, suggests that previous alcohol use is probably a risk factor but did not reach significance due to the modest sample size.

In contrast to the general population (in which the top three causes of death are ischaemic heart disease, cerebrovascular disease, and dementia) [16], and also in contrast to the Indigenous population of Australia as a whole (in which the top three causes of death are ischaemic heart disease, diabetes, and lung cancer) [28], the leading causes of mortality in this remote Aboriginal population were diabetes, renal failure, and ischaemic heart disease. Treating and preventing these conditions, particularly diabetes and renal failure, must be a priority for public health practitioners working with remote Indigenous communities in Western Australia.

Unlike type 1 diabetes, which is uncommon in Indigenous Australians, the prevalence of type 2 diabetes is very high [7]. Aboriginal peoples are diagnosed with diabetes at a younger age than non-Indigenous Australians (often in early adolescence), and die from it earlier [7, 29]. Wang et al. conducted a longitudinal study of 686 Indigenous Australians aged 20–74 years living in the Northern Territory to investigate the incidence of diabetes in this population over 13 years. Using hospital records, they estimated that by the age of 60 years, 49% of Indigenous men and 70% of Indigenous women had diabetes [30]. Probable explanations include the high prevalence of both childhood and adult obesity (in 2012–13, 30% of Indigenous children aged 2–14 years, and 66% of Indigenous people aged ≥15 years were overweight or obese) [2], low levels of physical activity (62% of Indigenous adults engage in low levels of, or no physical activity) [2], poor nutrition (which may be especially acute in remote Australia, where access to fresh and nutritious food is limited) [29], and smoking (41% of Indigenous people aged ≥15 years smoke—double the prevalence in the general population) [2]. Genetic factors (the “thrifty genotype”) and epigenetic factors may also play a role [31], particularly in conjunction with the loss of traditional lifestyles and associated change to a Western diet (shifting from consumption of lean indigenous animals to high-fat meats) [32]. Overweight mothers have a higher risk of gestational diabetes, and intrauterine exposure to hyperglycaemia increases the likelihood that affected children will develop obesity or diabetes themselves [32].

Preventing and treating diabetes appears vital, given its contribution to renal disease and ischaemic heart disease—the second and third leading causes of death in the cohort. Diabetic nephropathy is the leading cause of end-stage renal failure in Indigenous Australians, accounting for 45% of cases compared to 17% in the general population [7, 8]. However, Aboriginal peoples, particularly those living in remote areas, face additional challenges. Late referral for treatment is common, probably attributable to socioeconomic factors including limited access to health services [8, 33]. Health promotion programs will need to take into account the structural and socioeconomic barriers to health and well-being in this population [7].

To date, many health promotion programs targeting the health disparity experienced by Aboriginal peoples have had limited impact and/or sustainability [34]. However, programs that are community-driven and include Aboriginal peoples in an equitable partnership can work. Gracey and colleagues reported that an Aboriginal-led program in remote Western Australia increased physical activity, reduced body weight, and decreased HbA1c and lipid levels. Knowledge about healthy lifestyle behaviours was increased [35]. The program has since been adopted by an Aboriginal health service in an urban setting. Gracey et al. demonstrate that the risk factors for type 2 diabetes and renal disease are amenable to change. However, reducing premature mortality from diabetes and renal disease will likely require multiple and prolonged health promotion strategies that include all relevant stakeholders to be successful.

Strengths of our study include the high response fraction (94%), comprehensive assessment using culturally specific tools, follow-up of up to 9 years, involvement of Aboriginal research assistants and, theoretically, the complete capture of all endpoints via electronic record linkage (bar one participant who could not be linked). However, data linkage is performed via probabilistic matching. While numerous validation studies have shown the number of incorrect or missed links is extremely low [13], these studies are based on the general population. Whether linkage error is similar for Indigenous people, particularly those living in remote Australia, is uncertain. For a variety of reasons, identifiers such as name, address, and date of birth may be inconsistently reported and subject to greater variation among Indigenous Australians than non-Indigenous Australians [36]. Misclassification of the cause of death prior to coding and incorporation into the data linkage system may also have occurred, owing to limited access to medical services in remote Australia and possible lack of a detailed medical history. However, such limitations are inherent in a study of this nature. Other limitations include the relatively modest sample size (although the cost and difficulty of fieldwork makes this hard to overcome), and possibility of response and recall bias. The participation of Aboriginal research assistants hopefully minimised elements of the former (e.g., acquiescence bias). A further limitation of the study is the comparison between mortality in the general population (which includes all age groups), and this sample of people aged ≥45 years. Broad and leading cause of death data (in the manner presented in Tables 2 and 3) were unfortunately not available for the general population stratified by specific age groups. However, given that the leading causes of death in this sample were diabetes, renal failure, and ischaemic heart disease, we believe that a comparison between our sample and the general population is appropriate. The observed causes of death are conditions from which, even in Indigenous populations, the overwhelming majority of deaths occur in those aged ≥45 years. One would expect relatively few deaths to occur before this age, and even allowing for such deaths (which are more likely in the Indigenous, rather than non-Indigenous population), our key finding remains true: that far more remote-living Aboriginal people in the Kimberley region die from diabetes and renal failure than one would expect.

## Conclusions

Aboriginal people living in the remote Kimberley region of Western Australia are more likely to die from diabetes and renal failure than both their non-remote Indigenous counterparts, and the Australian population as a whole. The proportion of deaths attributable to other causes was similar to that of the general population. Together, diabetes and renal disease accounted for nearly one-third of mortality in this remote population of older Indigenous people. Given that the risk factors are known and amenable to change, reducing mortality from these preventable conditions is feasible and could substantially improve the mortality gap. However, a holistic and sustained effort will be required.

## Supporting information

S1 Fig Kaplan-Meier survival curves showing associations between age and all-cause mortality. (PNG)
