# Supplementary figures and images for: Mortality in a cohort of remote-living Aboriginal Australians and associated factors
Source: PLoS One. 2018 Apr 5;13(4):e0195030. doi: 10.1371/journal.pone.0195030 (PMC5886486; doi:10.1371/journal.pone.0195030)

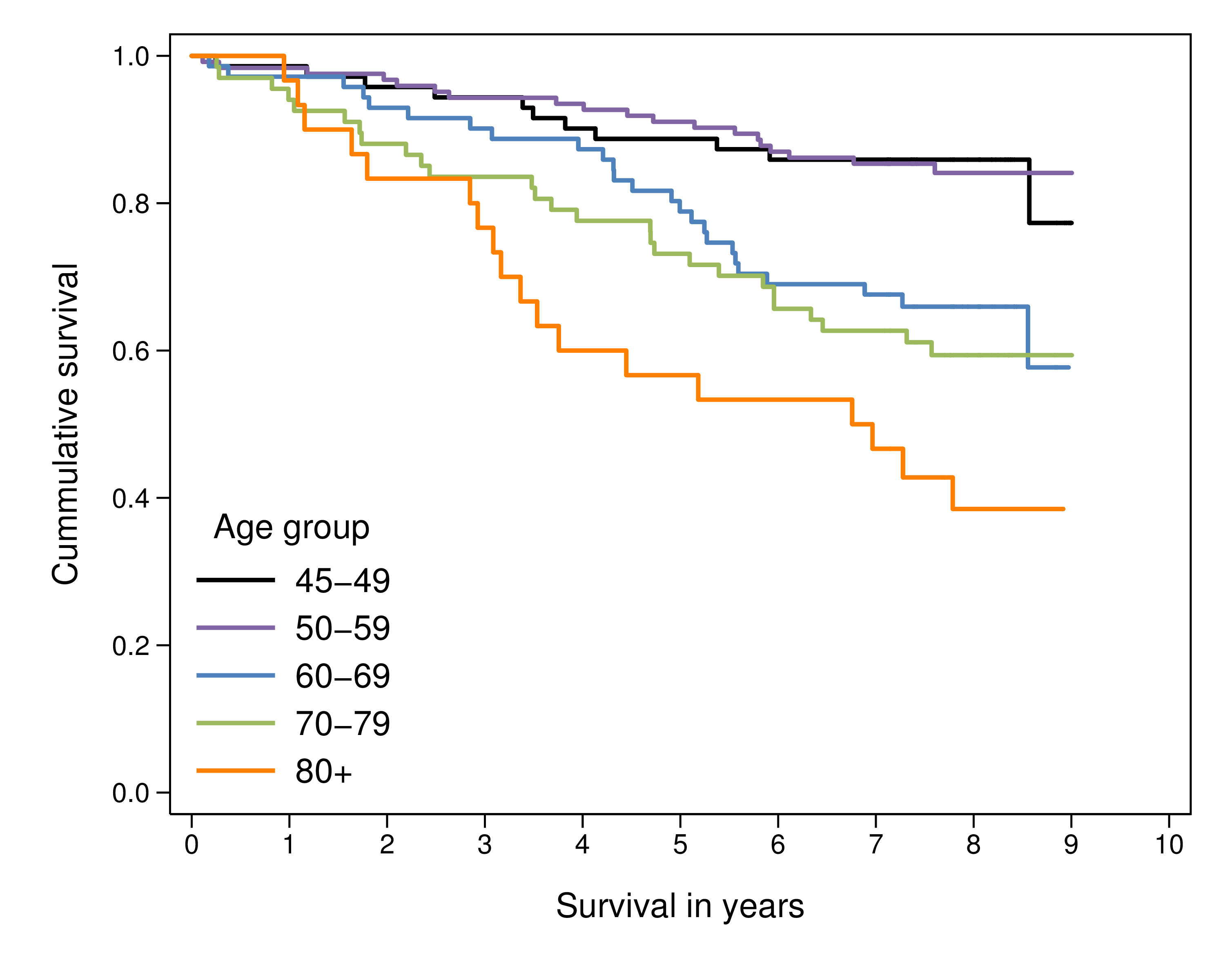

Supplement: S1 Fig — (PNG) [file pone.0195030.s001.png]
